# Double-Layer Sol–Gel Modifications on Titanium Alloy Substrates—Physicochemical Properties Evaluation

**DOI:** 10.3390/ma18163857

**Published:** 2025-08-18

**Authors:** Katarzyna Matysiak, Maria Biegun-Żurowska, Katarzyna Cholewa-Kowalska, Tomasz Goryczka, Wojciech Zając, Magdalena Ziąbka

**Affiliations:** 1 Department of Ceramics and Refractories, Faculty of Materials Science and Ceramics, AGH University of Krakow, 30-059 Krakow, Poland; biegun@agh.edu.pl (M.B.-Ż.); ziabka@agh.edu.pl (M.Z.); 2 Department of Glass Technology and Amorphous Coatings, Faculty of Materials Science and Ceramics, AGH University of Krakow, 30-059 Krakow, Poland; cholewa@agh.edu.pl; 3 Institute of Materials Science, University of Silesia in Katowice, 75 Pułku Piechoty 1A, 41-500 Chorzow, Poland; tomasz.goryczka@us.edu.pl; 4 Department of Hydrogen Energy, Faculty of Energy and Fuels, AGH University of Krakow, 30-059 Krakow, Poland; wojciech.zajac@agh.edu.pl

**Keywords:** TiAlV alloy, TiN, hBN, hybrid composite layers, sol–gel method

## Abstract

The objective of this study was to investigate the physicochemical properties of hybrid coatings with titanium nitride and boron nitride nanoparticles deposited on the TiAlV medical alloy via the sol–gel process. The developed layers were intended to impart bactericidal properties and provide protection against surgical abrasions during the implantation procedure. This study focused on evaluating the microstructure (SEM + EDS), structure (XRD, FTIR), and surface properties, including wettability, surface free energy, and roughness of the synthesized layers. Our results confirmed that it was feasible to produce hybrid layers with various microstructures and diverse layer morphologies. The FTIR and XRD structural analyses confirmed the presence of an organosilicon matrix incorporating the two aforementioned types of ceramic particles.

## 1. Introduction

Titanium and its alloys, specifically Ti6Al4V, have become widely applicable in biomedicine, especially in orthopedics and dentistry [1,2,3]. Titanium alloys display exceptional biocompatibility; they promote successful tissue integration, have a high strength-to-weight ratio, and feature a low modulus of elasticity [4,5]. Additionally, Ti alloys demonstrate superior mechanical properties, including high fatigue strength (140–1160 MPa) and fracture toughness [6,7].

Nevertheless, given the complex physiological conditions in the human body and the diverse treatment needs, there is a growing demand for higher performance from Ti6Al4V alloy [8,9,10,11]. Recent studies have confirmed that titanium alloy implants have adverse effects. They pose challenges, such as cytotoxicity stemming from the release of alloying elements, stress shielding related to a mismatch in the elastic modulus between bone and implant, bone resorption and, consequently, inadequate osseointegration [3,12,13,14]. It has been reported that titanium alloy implants may gradually release alloying elements such as aluminum (Al), vanadium (V), and other ions, which are under investigation for their potential to induce allergic reactions and to influence therapeutic outcomes in certain cases [3,15,16]. On the other hand, the strengthened antimicrobial properties of the Ti6Al4V alloy prevent bacterial infections after implantation, clinical complications and implantation failures, which pose serious health risks to patients and place an economic burden on healthcare systems. Therefore, it is crucial to research methods for enhancing both the corrosion resistance and antibacterial properties of titanium alloy surfaces in physiological environments [1,17].

In recent years, among various surface modification technologies in coatings, the sol–gel method has been frequently investigated as an environmentally friendly and inexpensive option [18,19,20,21,22]. The process involves forming an oxide network on the metal surface via initial hydrolysis and the subsequent condensation of alkoxy compounds. According to the literature, this type of coating results in metal substrates endowed with corrosion protection properties [18,23,24]. The sol–gel method may also serve as a pretreatment, enhancing adhesion between the metallic substrate and the final hybrid organic–inorganic coatings [25,26]. Furthermore, sol–gel coatings are generally applied at low temperatures, which supports incorporating various components into the silica matrix with no risk of thermal decomposition [21,27]. The dip coating technique has numerous advantages over other methods. It is more cost-effective and requires fewer instruments. Additionally, it is possible to coat both sides of substrates, cover larger areas, and control the coating structure more effectively. Several parameters influence the properties of dip-coated films, e.g., the concentration and viscosity of the coating solution, withdrawal speed, number of dips, and thermal treatment. These factors affect the thickness and morphology of the film [28].

It has been reported that various ceramic nanoparticles, such as SiO_2_, TiN, and hBN, can be incorporated into different matrices via the sol–gel process so as to obtain hydrophobic functional coatings [25,29,30,31,32,33]. Nitride particles are characterized by remarkable hardness, chemical inertness, oxidation resistance, mechanical strength, interface lubricity, high electrical and heat resistance, and low density [31,34,35,36]. They have already been tested for use as dental cement, and in other medical and cosmetic applications [35,37]. Hexagonal boron nitride and titanium nitride are used as coatings in a variety of applications [19,36,37]. They are common in biomedical applications due to their structural similarity to graphene. For instance, thin films doped with hBN or TiN nanoparticles are beneficial for bone implants, as they reduce associated infections [28,38,39]. Therefore, hBN or TiN nanoparticles might improve bone recovery and osseointegration [28,40,41].

In the present work, three types of multi-layer hybrid coatings composed of organically modified silica and titanium precursors, enriched with titanium nitride or boron nitride nanoparticles, were developed through the sol–gel dip coating. In each layer, the proportion of ceramic nanoparticles was kept constant at 1.25% molar. The structural and surface properties of the coatings were assessed using different techniques. The microstructure and chemical compositions were examined via SEM-EDS, and the structure via XRD and FTIR. The mechanical properties (hardness and adhesion strength) and surface properties (roughness and wettability) were investigated as well. 

## 2. Materials and Methods

### 2.1. Metal Substrate Preparation

Titanium alloy plates (Ti6Al4V, 25 × 15 mm) supplied by Wolften (Wrocław, Poland) were subjected to either a two-step or three-step cleaning procedure. In both cases, the samples were first sonicated in acetone for 30 min, followed by sonication in ethanol for an additional 30 min. For the three-step protocol, the final step involved etching half of the plates in 5% hydrofluoric acid (HF) for 30 s. After each stage, the substrates were rinsed thoroughly with deionized water.

### 2.2. Preparation of SiO_2_/BN and SiO_2_/TiN Samples

#### 2.2.1. Synthesis of Hybrid Sols

**Figure 1:**
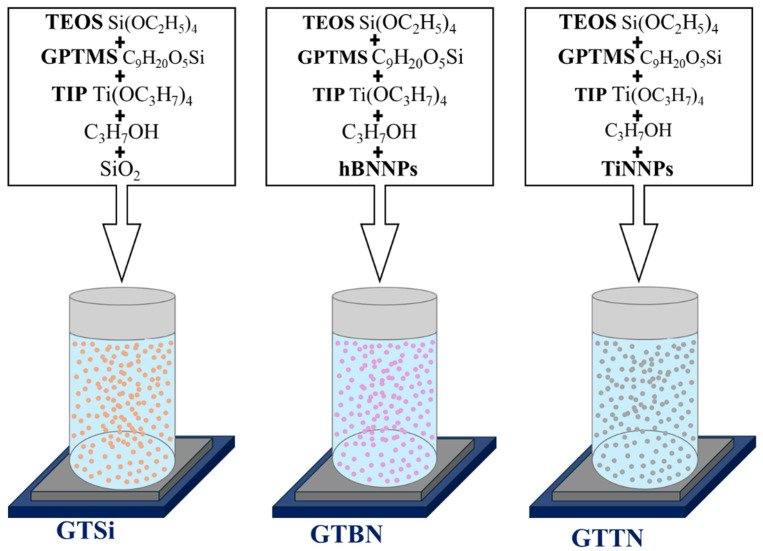
Chemical compositions of obtained hybrid sols GTSi, GTBN, and GTTN.

Three hybrid sol solutions were formulated, with their chemical compositions detailed in Figure 1. These solutions were prepared at room temperature by mixing titanium (IV) isopropoxide (TIP, Aldrich (Melbourne, Australia), 97%), tetraethoxysilane (TEOS, Aldrich, 99%), and 3-(glycidyloxypropyl) trimethoxysilane (GPTMS, Aldrich, 98%) using propanol (POCH Basic (Gliwice, Poland), 99.7%) as a solvent. Following 30 min of stirring, 0.2 mL of concentrated hydrochloric acid, serving as a catalyst, was incorporated into 20 mL of the mixture. A suitable amount of colloidal silica (SiO_2_) suspension was added to the first sol (GTSi) to achieve a molar ratio of GPTMS/TEOS/TIP/SiO_2_ equaling 0.33/0.6/0.05/0.02. The preparation of the subsequent solutions, GTBN (1) and GTTN (2), followed the same methodology, but utilized boron nitride (1) and titanium nitride (2) nanoparticles (sourced from Sigma-Aldrich, Saint Louis, MO, USA) instead of the SiO_2_ suspension. The average hydrodynamic diameter of the TiN nanoparticles was approximately 178 nm, while for the hBN nanoparticles it was around 230 nm (DLS measurement—Supplement File Figure S1). Prior to their addition, the nitride nanoparticles were ultrasonically dispersed in propanol for 45 min, then incorporated into the solutions before the catalyst (HCl) was introduced. The component quantities were selected to maintain a ceramic particle concentration of 1.25% molar. This concentration was chosen to ensure that the nanoparticle suspension remained stable and homogeneous for at least 48 h. The resulting solutions were aged for 24 h and stored in a sealed glass container.

#### 2.2.2. Coatings Deposition

**Figure 2:**
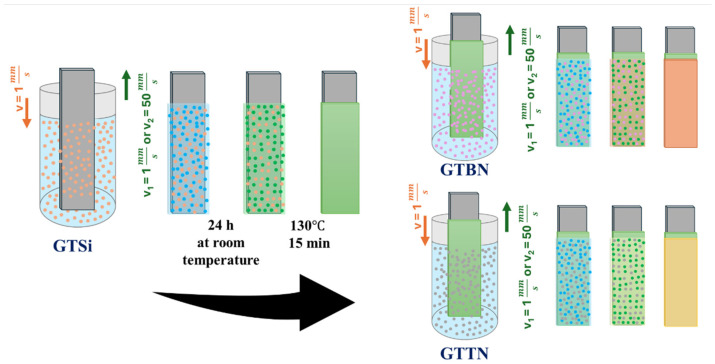
The general procedure of sample preparation with sol–gel layers.

Double-layer sol–gel systems were the combination of GTTSi and GTBN/GTTN sols deposited via dip coating with a fully automated dip coater at two different withdrawal speeds, v_1_ = 1 mm/s and v_2_ = 50 mm/s. The schematic coating deposition and curing are presented in Figure 2. The first layer was dried under ambient conditions for 24 h, then stabilized through a two-step thermal treatment at 80 °C for 10 min and 130 °C for 15 min. The same procedure was followed after the second layer was applied.

The following nomenclature was adopted to standardize the names of the samples (Table 1). The research results were compared with two reference samples: a pure metal alloy without any applied layers (TiAlV) and a sample that was etched with hydrofluoric acid (TiAlVHF).

### 2.3. Coatings Characterization

#### 2.3.1. Scanning Electron Microscopy

The SEM-EDS investigations were carried out using the Apreo 2S high-resolution scanning electron microscope from ThermoFisher Scientific (Waltham, MA, USA). Depending on the sample, the observations were performed using either the in-column detector T1, the low vacuum detector (LVD) from ThermoFisher Scientific (Waltham, MA, USA) or the Everhart–Thornley Detector (ETD) at an accelerating voltage of 5–10 kV. The qualitative and quantitative analyses were performed using a standardless method with the SDD detector, specifically the EDAX Octane Elite, operated with APEX™ Advanced 2022 software, version 2.5.1001.001. The qualitative analysis was performed using a standardless method.

#### 2.3.2. Fourier Transform Infrared Spectroscopy (FTIR)

The BTS-RAD FTS 3000 Excalibur spectrophotometer (Bio-Rad, Hertfordshire, UK) was employed to examine the structural changes caused by chemical modifications of the substrates with the sol–gel layers. The ATR technique, utilizing the MIRACLE attachment with a ZnSe-doped diamond crystal, was used. The analyses were conducted over a range of 400–4000 cm^−1^ with a resolution of 4 cm^−1^.

#### 2.3.3. XRD X-Ray Diffractometry

The X-ray diffraction (XRD) patterns were measured using the X’Pert Pro PANalytical diffractometer (Malvern Panalytical Ltd., Malvern, UK). The instrument was equipped with a copper X-ray tube and beam geometry that emitted monochromatic radiation at the wavelengths of 0.1530598 nm (CuKα1 line) and 0.1544938 nm (CuKα2 line). Two X-ray diffraction techniques were used: the classical Bragg–Brentano (BB) and X-ray grazing incidence beam (GIXD). Depending on the technique, the diffractograms were measured by step-scan in the 2θ ranges of 5–140° (BB) and 5–100° (GIXD). The step and measurement time were optimized to ensure adequate counting statistics.

#### 2.3.4. Roughness

The arithmetical mean roughness (Ra) and the root mean square roughness (Rq) of the materials were measured using the contact profilometer HOMMEL-ETAMIC T1000 wave (Jenoptik AG, Jena, Germany). The Ra values were calculated as an average of 10 measurements, each with a traverse length of 4.8 mm, following the EN ISO 4288 standard [42]. The results are presented as the mean ± standard deviation (SD).

#### 2.3.5. Surface Wettability

The static water contact angle (WCA) was measured to evaluate surface wettability, using the sessile drop method with the DSA 10 Mk2 automatic drop shape analysis system (Kruss GmbH, Hamburg, Germany). The tests were conducted under constant temperature and humidity conditions. UHQ-water droplets of 0.25 μL were applied to each clean and dry sample. The apparent contact angle was calculated as an average of 30 measurements and reported as the mean ± standard deviation (SD).

#### 2.3.6. Surface Free Energy

The free surface energy was determined by measuring the contact angles of two liquids: ultra-pure distilled water (UHQ PURE Lab., Vivendi Water, Paris, France) and diiodomethane. The measurements were performed using the DSA 10 Mk2 optical apparatus (Kruss GmbH, Hamburg, Germany) at room temperature. The testing procedure followed the same pattern as the contact angle test. The photographs of the droplets from the two liquids were used to calculate free surface energy based on the dispersion and polar components values. The final value represents an arithmetic average of 30 measurements, with the results presented as the mean ± standard deviation (SD).

#### 2.3.7. Mechanical Properties

To assess the coatings’ microhardness, Vickers microhardness measurements were performed, using the FM700 hardness testing machine (Future Tech, Corp., New York, NY, USA) with an applied load of 0.25 N. The tests were conducted at room temperature under atmospheric conditions, and the reported values represent an arithmetic average of 10 measurements. The results are presented as the mean ± standard deviation (SD). The mechanical properties were evaluated with the ZwickRoell testing machine (ZwickRoell, Ulm, Germany). The adhesion strength was measured during a tensile test at a test speed of 2 mm/min according to EN 4624.ZP2 standards [43] on 4 samples of each material. The results are expressed as the mean ± standard deviation (SD).

#### 2.3.8. Corrosion Test

Electrochemical Impedance Spectroscopy (EIS) tests were performed to evaluate the corrosion behavior of metallic substrates coated with protective layers. The tests were conducted using a three-electrode electrochemical cell using Solartron 1252 frequency response analyzer coupled with Solartron 1287 potentiostat (Ametek, Leicester, UK) in a frequency range of 0.24 Hz–300 kHz with an excitation voltage of 10 mV (rms). The coated samples served as the working electrodes, exposing a flat, circular surface area of 0.8 cm^2^ to the electrolyte. A platinum grid was used as the counter electrode, and a 3M Ag/AgCl/KCl (SSCE) electrode was used as the reference. All potentials reported in this study are referenced to SSCE. All electrochemical tests were conducted in a 0.9% NaCl solution at 37 °C, in accordance with general guidance from ISO 10993-15 [44]. Prior to testing, all the samples were incubated in the electrolyte for 24 h at 37 °C to allow surface stabilization and solution penetration into potential coating defects. The measurements were repeated at least twice for each material to ensure reproducibility.

## 3. Results and Discussion

**Figure 3:**
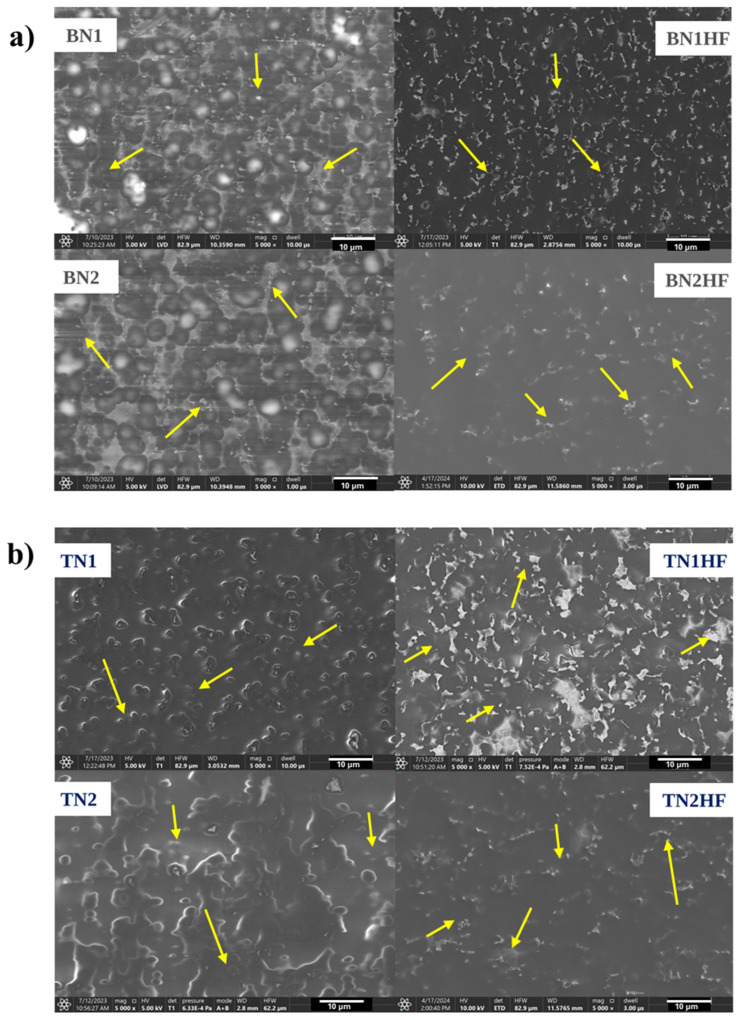
SEM microphotographs of SiO_2_ coatings deposited on TiAlV samples and obtained in sol–gel reaction containing hBNNPs (**a**) and TiNNPs (**b**).

**Figure 4:**
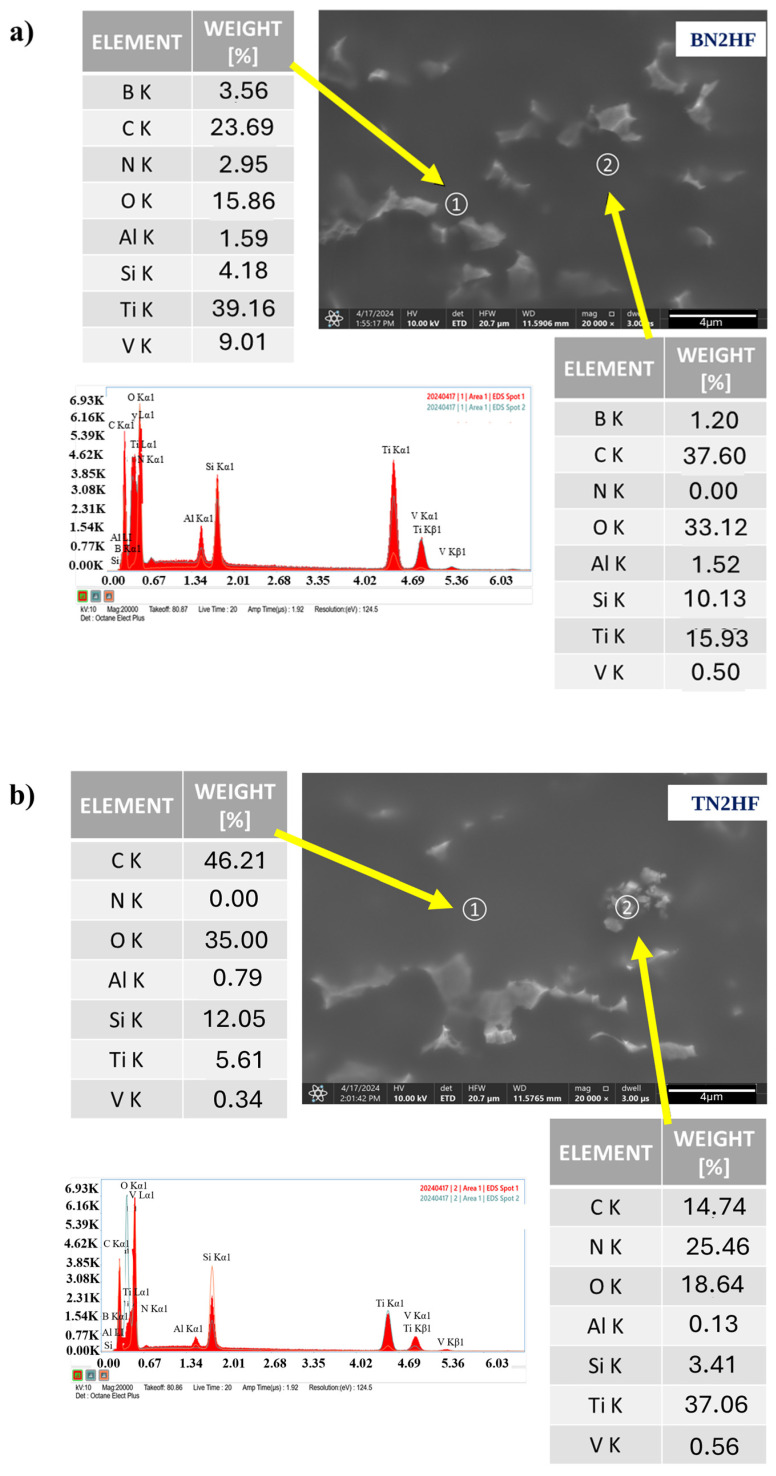
SEM images and EDS spectra of titanium alloy (TiAlV)-covered sol–gel hybrid layer containing hBNNPs (**a**) and TiNNPs (**b**).

Figure 3 shows SEM images of the silica coatings deposited on the Ti6Al4V alloy substrate with the incorporated TiN and hBN particles. The surface observations confirmed the presence of the ceramic particles on the modified titanium alloy surface (marked with yellow arrows in the images). The nanoparticles’ distribution is presented in the Supplement File (Figure S1). However, the results also indicated the layers were not completely homogeneous, as nitride particles with an uneven distribution were revealed. Neither pores nor obvious cracks were observed in the layer. The presence of nitride nanoparticles was further confirmed through the EDS chemical analysis conducted for the BN2HF and TN2HF samples. The layers appeared continuous at the 10,000× magnification, although on the surface there were clearly visible agglomerates (Figure 4). The chemical analysis at point 1 displayed the BN particles of about 0.3 µm, while at point 2 the analysis confirmed the presence of an organic–inorganic matrix (Figure 4a). In the case of layers with TiN nanoparticles measuring 0.5 µm (Figure 4b), point 1 enabled the layer identification and point 2 confirmed the particles’ presence. However, the chemical composition given in the tables is indicative and does not represent an analysis of a precise amount.

**Figure 5:**
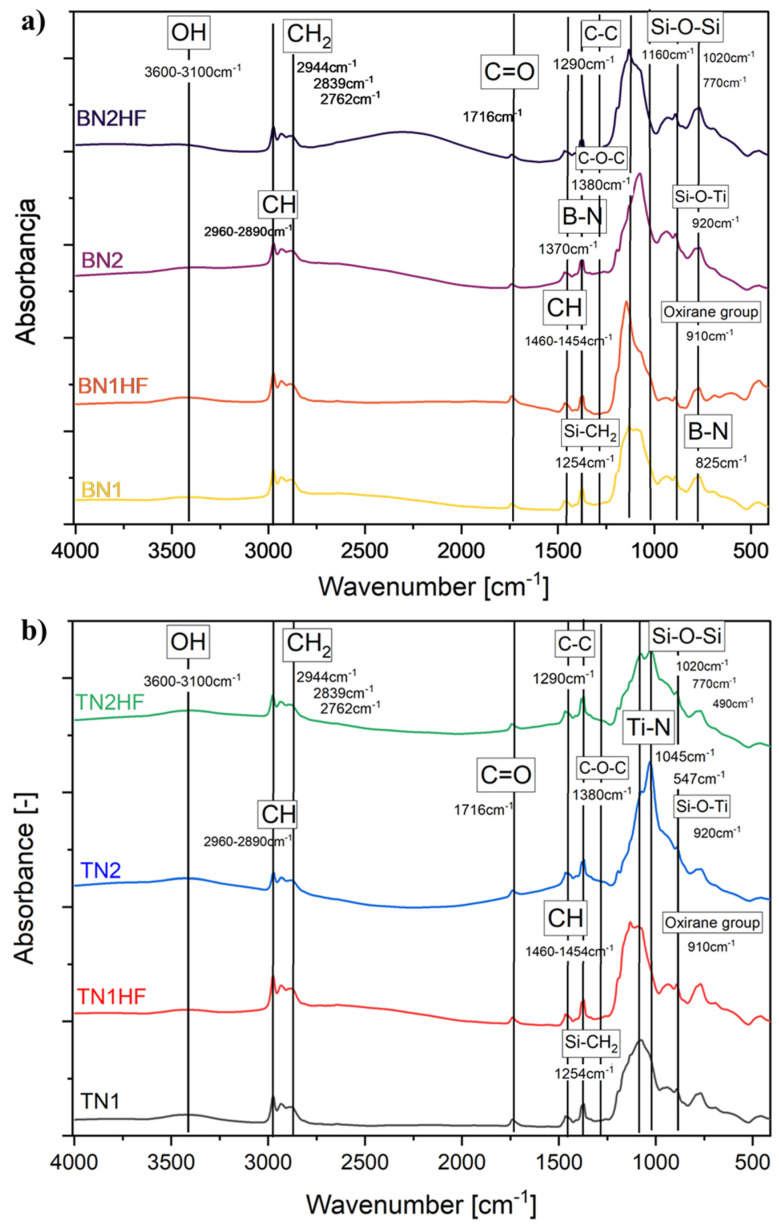
FTIR spectra of titanium alloy (TiAlV)-covered sol–gel hybrid layer containing 1.25 mol% hBNNPs (**a**) and TiNNPs (**b**).

Figure 5 shows the FTIR spectra of the samples immediately after the layer application in the 400–4000 cm^−1^ region. In all the spectra, the weak band observed in the spectral range 3100–3600 cm^−1^ was attributed to the physically and/or chemically bound water, specifically representing the symmetric OH stretching of asymmetrically hydrogen-bonded water molecules. Additionally, the CH alkyl stretching bands were detected in the 2890–2960 cm^−1^ region [45,46]. The C-H bending vibrations were associated with a band observed in the 1454–1460 cm^−1^ region, while those at 1380 and 1290 cm^−1^ were attributed to the skeletal vibrations of C-O-C derived from GPTMS [47]. The bands at 770, 1020, and 1160 cm^−1^ related to the stretching vibration of Si-O-Si bonds [48,49]. The appearance of these bands verified the polycondensation reaction between TEOS and GPTMS in the coating [25]. A weak band at 910 cm^−1^ showed the presence of the epoxy ring from GPTMS [50]. Moreover, a weak band at 955 cm^−1^ was attributed to Ti-O-Si vibration [51]. A weak and broad band at 795 cm^−1^ corresponded to the Si-C bonds of the CH3SiO_3_ groups on the surfaces of silica particles. This peak resulted from the condensation between Si-OH and GPTMS, which accounted for the increased hydrophobicity of the coating [48,52]. The FTIR spectrum of the coatings with hBN particles (Figure 5a) demonstrated characteristic bands at 825 cm^−1^ and 1370 cm^−1^ attributable to the B-N bond vibrations. Specifically, the strong and wide band at 1370 cm^−1^ corresponded to the in-plane B-N stretching vibration, while the narrower band at 825 cm^−1^ was linked to the out-of-plane bending vibration of B-N-B [53,54,55]. In Figure 5b, the band at 1045 cm^−1^ could be attributed to Ti-N stretching vibrations, while vibrations at 547 cm^−1^ could be assigned to Ti-N stretching modes [56,57].

**Figure 6:**
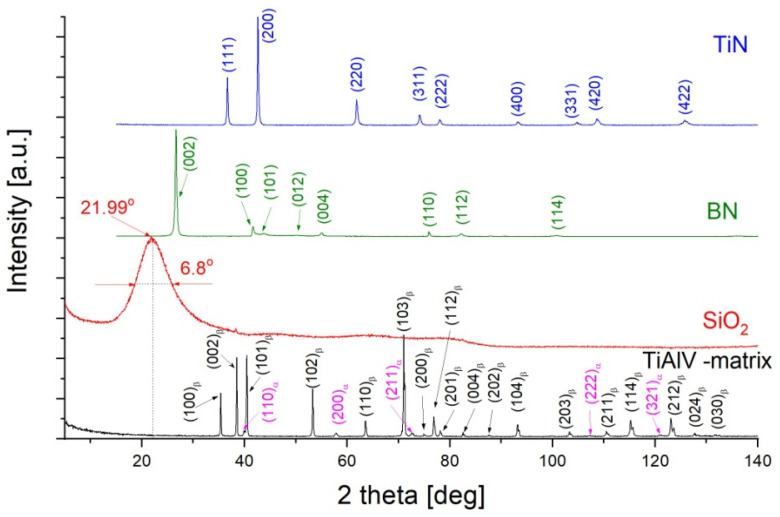
X-ray diffraction patterns measured for composite components before layer deposition.

Prior to the X-ray phase analysis, the starting substance powders were characterized in terms of their phase composition. The measured diffractograms in classical Bragg–Brentano geometry are presented in Figure 6. The metallic substrate consisted of two phases—the polymorphic β-TiAlV variant (hcp structure—with the P63/mmc space group) with a predominant volume fraction and α-TiAlV (bcc structure—with the Im3m space group). The measured diffractogram for the matrix is presented as a black line in Figure 6. A common component used in the BN and TiN deposits was SiO_2_ used as a water suspension (Figure 5—red line). The measured diffractogram revealed only one line with a half-width of approximately 6.8 degrees. The peak broadening proved the SiO_2_ phase to be an amorphous state. The position of this peak—21.99° (2theta) corresponded to an interplanar distance of 4.038 Å. This distance is characteristic of the strongest diffraction line of SiO_2_ in the polymorphic form of cristobalite (ICDD PDF-4 card no 00-039-1425). The other two components, boron nitride and titanium nitride, were crystalline. The measured diffractograms are marked in Figure 6 as green and blue lines for the BN (ICDD PDF-4 card no. 01-073-2095) and TiN (ICDD PDF-4 card no. 00-038-1420), respectively.

**Figure 7:**
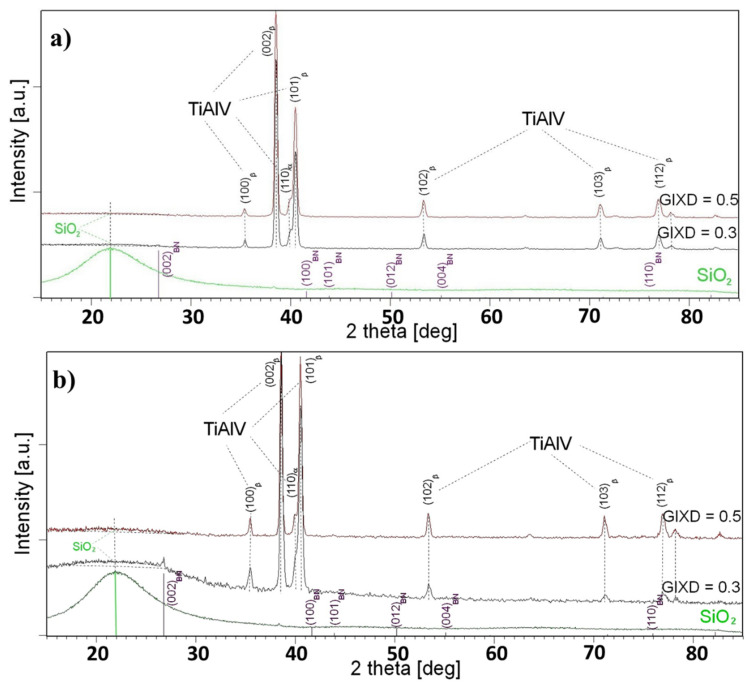
GIXD patterns measured for BN1 (**a**) and BN2 (**b**) coatings deposited on non-etched TiAlV matrix.

**Figure 8:**
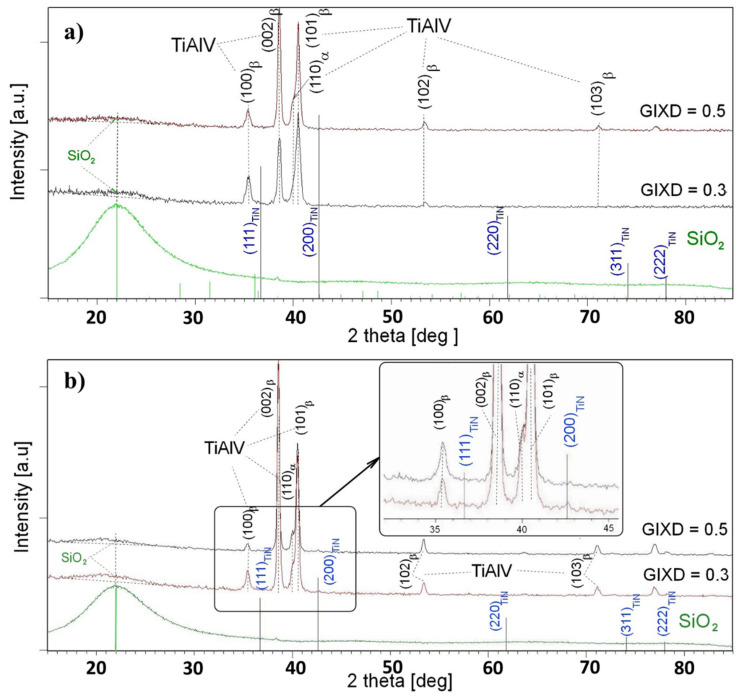
GIXD patterns measured for TiN1 (**a**) and TiN2 (**b**) coatings deposited on non-etched TiAlV matrix.

The diffractograms obtained in the geometry of a constant grazing incidence angle (GIXD) were used to identify the phase of the produced layers. This technique provides structural information from small near-surface depths, where the volume fraction of the layer material increases. As a result, the diffraction lines characteristic of the material forming the layer appeared in the diffraction patterns (Figure 7 and Figure 8). Since the etching of the TiAlV substrate did not affect the layer formation, the results are discussed for the layers deposited on the non-etched substrate. These samples were marked as BN1, BN2, TiN1, and TiN2, with the symbols referring to their withdrawal speeds (Table 1).

Figure 6 shows the diffractograms measured at constant angles of 0.3° and 0.5° for the boron nitride layers that were deposited on the non-etched TiAlV substrate with withdrawal speeds of 1 and 50 mm/s—Figure 7a and Figure 7b, respectively. For comparison, they were contrasted with the diffractogram measured for SiO_2_ (green line) and the theoretical positions of the diffraction lines of the boron nitride standard (blue bars). Regardless of the deposit type applied to the TiAV matrix, the layers produced at a speed of 1 mm/s resulted in the diffractograms showing only lines representing the crystalline phases characteristic for the substrate: β-TiAlV and α-TiAlV. Moreover, a low-intensity peak was found in the 2theta position, characteristic of the amorphous SiO_2_ phase. This fact proved that the layer was composed only of SiO_2_, while the areas containing boron nitride (BN1) did not exceed the X-ray detection limit. Hence, no lines characteristic of this phase were observed in the diffraction patterns. A similar situation occurred in the case of the TiN1 sample for the layer of titanium nitride (Figure 8a).

Increasing the withdrawal speed to 50 mm/s contributed to the formation of a thin amorphous SiO_2_ layer on both the BN2 and TiN2 layers. Moreover, the GIXD diffraction pattern measured for the BN2 (constant angle of 0.3°) revealed diffraction lines mainly coming from the matrix. Additionally, the strongest diffraction line (002) characteristic of the BN phase occurred (Figure 7a). In the case of the TiN2 sample, the diffraction pattern revealed two diffraction lines (111) and (200) belonging to the TiN lines with the strongest intensity.

**Figure 9:**
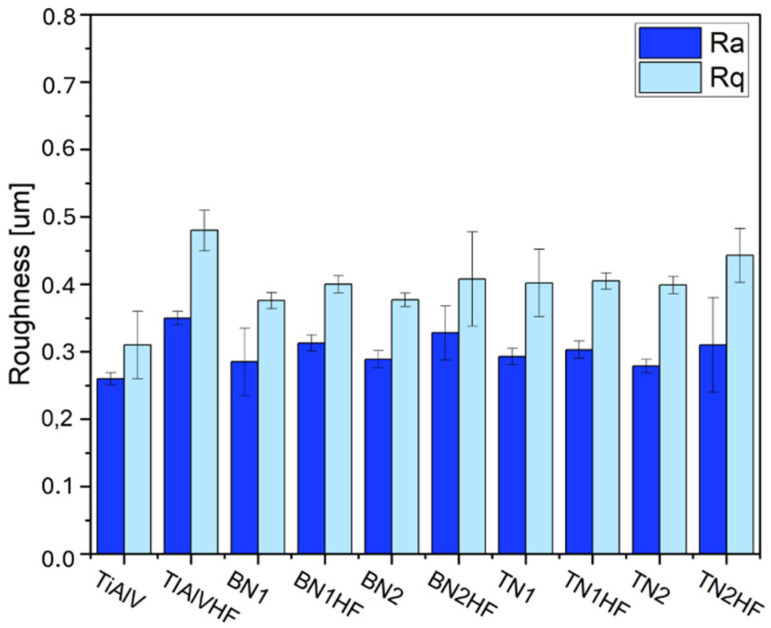
Roughness parameters of titanium alloy (TiAlV)-covered sol–gel hybrid layer containing ceramic particles.

The measurements revealed that the TiAlV alloy modified with the sol–gel layers was noticeably enhanced. The increase in the Ra and Rq parameters was observed for all the tested layers in comparison to the base alloy (Figure 9). It is evident that etching the Ti6Al4V alloy prior to the deposition process increased the surface microroughness, as was proven by the Ra and Rq elevation [58,59]. The Ra parameter rose by 0.10 µm on average, and the Rq parameter by 0.18 µm. Etching not only eliminated adverse surface impurities but also changed the surface topography [58,60,61,62]. Introducing ceramic particles into the layers reduced roughness parameters in comparison to the etched alloy (TiAlVHF sample). The reduced roughness of the layers containing TiN and BN nanoparticles resulted from the particle size within the layer and the particles’ uneven distribution, which correlated with the SEM observations.

**Figure 10:**
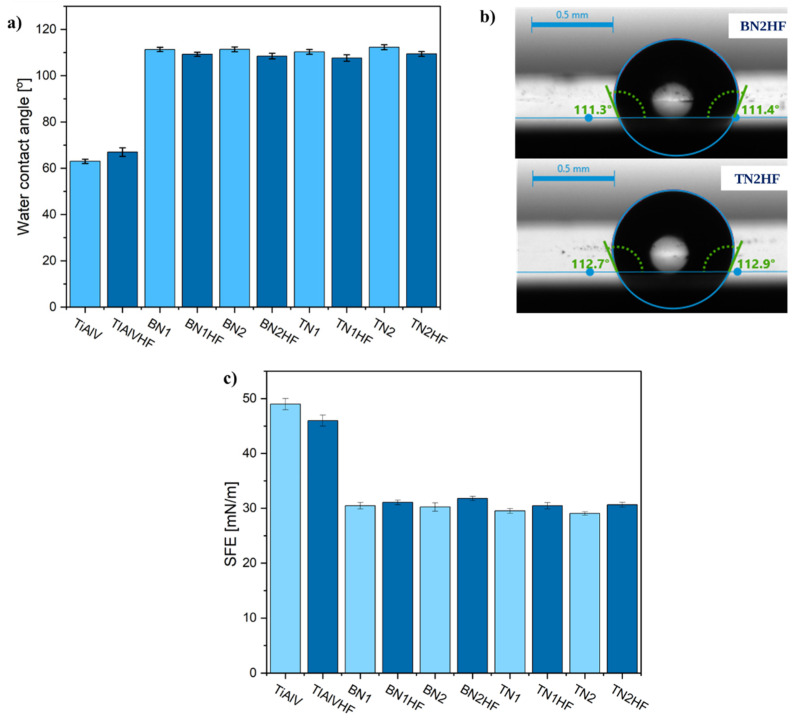
Static water contact angle (**a**), corresponding images (**b**) and surface free energy (**c**) of titanium alloy (TiAlV)-covered sol–gel hybrid layer containing ceramic particles.

The modification of TiAlV alloys with organic–inorganic layers had a pronounced effect on their surface characteristics, as evidenced by the measurements of the contact angle (Figure 10a) and surface free energy (Figure 10c). The application of hybrid coatings significantly increased the contact angle while reducing the surface free energy, with both parameters strongly influenced by the ceramic particle content. For the BN2HF layers, the contact angle increased by an average of 50°, accompanied by a 19.6 mN/m decrease in surface energy. Similarly, the TN2HF layers exhibited an average contact angle increase of 49°, with an 18.9 mN/m reduction in energy. These results indicate a clear shift from hydrophilic to hydrophobic surface behavior (Figure 10b). The better hydrophobicity of the enriched coatings may be attributed to the elevated surface roughness, consistent with the Wenzel and Cassie–Baxter models, which relate roughness to wettability [29,63,64].

**Figure 11:**
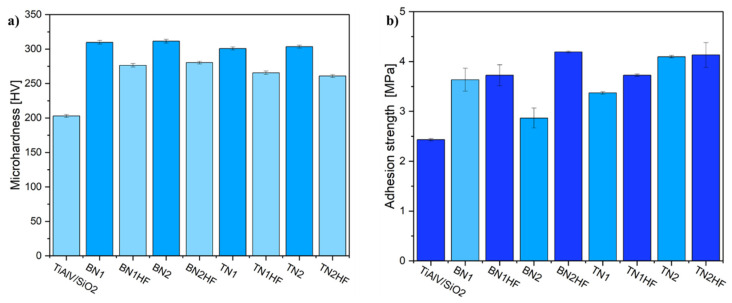
Microhardness (**a**) and adhesion (**b**) measured for samples coated with sol–gel containing ceramic particles.

Figure 11a illustrates the microhardness changes of the measured samples. The plot analysis revealed that the addition of ceramic particles significantly increased microhardness in comparison to the base layer (TiAlV/SiO_2_). The hardest samples were the ones whose surfaces had not been etched. Regrettably, none of the developed layers attained the microhardness level of the starting alloy, i.e., exceeding 360 HV [65]. In further studies, the adhesion of the layers to the titanium substrate was examined by measuring the pull-off strengths. The results are summarized in Figure 11b. The average pull-off strength was 2.4 MPa for the TiAlV/SiO_2_ reference. In general, the proposed layers adhered more effectively to the etched metallic substrate, which stayed in accordance with the literature [66,67]. After the incorporation of the hBNNPs or TiNNPs, the BN2HF specimen revealed that the pull-off strength increased by 71% (to 4.1 MPa) and the highest value was obtained for TN2HF (4.2 MPa). However, the resulting films’ adhesion was marginally lower compared to the other sol–gel coatings; this difference may be attributed to the ceramic nanoparticle size utilized in the preparation [68,69]. Still, it is worth noting that the interface adhesion measured via this method is limited by the adhesive strength, which is usually lower than 80 MPa [68].

**Figure 12:**
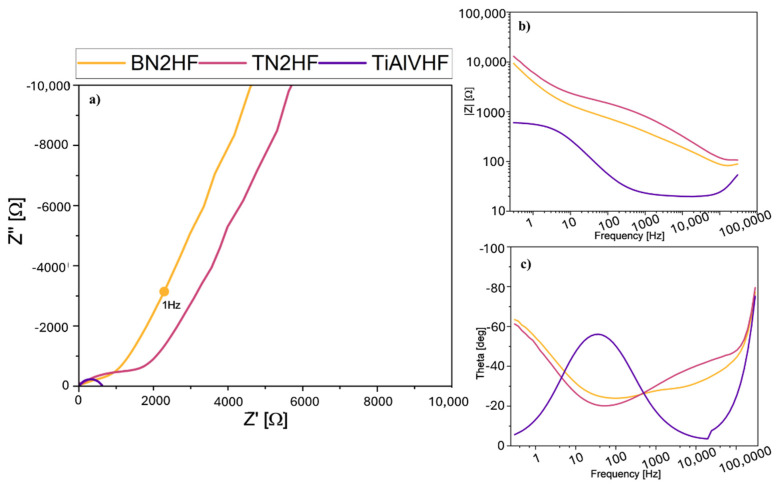
Electrochemical Impedance Spectroscopy (EIS) results for TN2HF, BN2HF, and TiAlVHF coatings in 0.9% NaCl solution at 37 °C: (**a**) Nyquist plots; (**b**) Bode plots showing the impedance modulus |Z| vs. frequency; (**c**) Bode plots showing the phase angle vs. frequency.

Beyond satisfactory adhesion performance, the coated samples that exhibited the best adhesion were further evaluated for their corrosion resistance. EIS measurements (Figure 12) revealed distinct differences in barrier effectiveness, with TN2HF exhibiting the highest impedance values, suggesting superior electrochemical protection. Electrochemical Impedance Spectroscopy (EIS) was employed to investigate the corrosion behavior of coated samples TN2HF, BN2HF, and TiAlVHF in a 0.9% NaCl solution at 37 °C. The Nyquist and Bode plots are presented in Figure 12. The Nyquist plots (Figure 12a) show depressed semicircles for all samples, indicative of capacitive behavior typical for coated metallic surfaces. In the case of the coated surfaces, the semicircle was followed by a straight line inclined at a sharp angle demonstrating blocking behavior of the coating. Among the tested samples, TN2HF exhibited the largest semicircle diameter, corresponding to the highest charge transfer resistance and, thus, the most effective corrosion protection. BN2HF presented an intermediate response, while TiAlVHF exhibited the smallest diameter, suggesting lower polarization resistance and inferior protective properties. The Bode magnitude plots (Figure 12b) further support this trend. TN2HF demonstrated the highest impedance modulus over the entire frequency range, indicating excellent barrier characteristics. BN2HF showed moderately high |Z| values, whereas TiAlVHF displayed the lowest, suggesting that the coating on TiAlVHF was the least resistive to ionic transport and corrosion processes. The phase angle plots (Figure 12c) highlight differences in coating behavior across the frequency spectrum. TN2HF maintained a broad phase maximum at intermediate frequencies, suggesting a more homogeneous and capacitive coating. In contrast, the TiAlVHF sample exhibited a narrower phase angle peak, indicative of more complex or defective electrochemical behavior. In conclusion, these EIS results confirm that the TN2HF coating provides the best corrosion resistance under simulated physiological conditions. The combination of high |Z| value, large phase angle, and extended low-frequency response suggest the presence of a dense, stable, and electrically insulating surface layer. In relation to the findings of other authors, Zang L. et al. [70] showed that the TiN nitride layer has higher charge transfer resistance and lower capacitance, which can effectively hinder the penetration and migration of reactive ions. Thus, the corrosion resistance of Ti6Al4V was significantly improved.

## 4. Conclusions

The microscopic studies confirmed the hybrid layers to be endowed with diverse microstructures and various layer morphologies. The layers produced via the sol–gel technique varied, depending on the applied precursor. The layer surface observations revealed that the distribution of nitride particles was not homogeneous. The coatings’ thickness was influenced by the withdrawal speed and the substrate preparation method. The non-etched samples were coated less efficiently. For the etched samples, enhanced adhesion of the coating to the substrate was obtained, accompanied by a slight reduction in micro-hardness. The FTIR and XRD structural analyses confirmed the presence of both the silicon matrix and the introduced ceramic nanoparticles. The modification of the Ti6Al4V alloy layer altered its surface properties, such as wettability, surface energy, and roughness. The TiN or hBN nanoparticles’ incorporation into the layers increased the contact angle values, which in turn lowered the surface energy. The modification of the TiAlV alloy with the ceramic particles sol–gel layers significantly augmented the surface roughness. The Ra and Rq parameter increase was observed for all the tested layers in relation to the base alloy. The examinations proved that applying a two-layer sol–gel coating to the alloy surface could effectively protect it from surgical scratches. Among all the investigated variants, the samples labeled BN2HF and TN2HF demonstrated the most favorable combination of physicochemical properties and exhibited satisfactory corrosion resistance. Therefore, these two coatings will be the focus of our future biological performance evaluation. In our next study, we will focus on the bactericidal properties and biocompatibility of the developed layers.

## Figures and Tables

**Table 1:** The sample nomenclature.

Sol 1	Sol 2	Withdrawal Speed $\left[\frac{mm}{s}\right]$	Sample Surface Preparation	Nomenclature of Samples
GTSi	GTBN	1	non-etched	**BN1**
	GTBN	1	etched	**BN1HF**
	GTBN	50	non-etched	**BN2**
	GTBN	50	etched	**BN2HF**
	GTTN	1	non-etched	**TN1**
	GTTN	1	etched	**TN1HF**
	GTTN	50	non-etched	**TN2**
	GTTN	50	etched	**TN2HF**

## Data Availability

The original contributions presented in this study are included in the article/Supplementary Material. Further inquiries can be directed to the corresponding author(s).

## Supplementary Materials

The following supporting information can be downloaded at: https://www.mdpi.com/article/10.3390/ma18163857/s1, Figure S1: Dynamic Light Scattering (DLS) analysis showing particle size distribution by number for: (a) hBN and (b) TiN powder.
