# Supplementary material for: Double-Layer Sol–Gel Modifications on Titanium Alloy Substrates—Physicochemical Properties Evaluation
Source: Materials (Basel). 2025 Aug 18;18(16):3857. doi: 10.3390/ma18163857 (PMC12387934; doi:10.3390/ma18163857)
Supplement: Supplementary file 1 [file materials-18-03857-s001.zip › materials-3776787-supplementary.pdf]

Supplementary Information

Double-layer Sol-Gel Modifications On Titanium Alloy Substrates - Physicochemical Properties Evaluation

Katarzyna Matysiak [1,*], Maria Biegun-Żurowska [1], Katarzyna Cholewa-Kowalska [2], Tomasz Goryczka [3], Wojciech Zając [4] and Magdalena Ziąbka [1]

[1] Department of Ceramics and Refractories, Faculty of Materials Science and Ceramics, AGH University of Krakow, 30-059 Krakow, Poland; biegun@agh.edu.pl; ziabka@agh.edu.pl

[2] Department of Glass Technology and Amorphous Coatings, Faculty of Materials Science and Ceramics, AGH University of Krakow, 30-059 Krakow, Poland; cholewa@agh.edu.pl

[3] Institute of Materials Science, University of Silesia in Katowice, 75 Pułku Piechoty 1A, 41-500 Chorzow, Poland; tomasz.goryczka@us.edu.pl

[4] Department of Hydrogen Energy, Faculty of Energy and Fuels, AGH University of Krakow, 30-059 Krakow, Poland; wojciech.zajac@agh.edu.pl

* Correspondence: kmatysiak@agh.edu.pl, Tel.: 691-626-118

1. Particle Size Distribution Analysis (DLS)

The particle size distribution of the hBN and TiN samples was analyzed using Dynamic Light Scattering (DLS). The results are presented in Figure S1.

For the hBN powder (Figure S1a), the Z-average diameter was 230.3 nm, with an exceptionally low polydispersity index (PDI = 0.012), indicating a highly monodisperse particle population. The dominant size peak was observed at 205.0 nm, and the standard deviation was 51.59 nm. The quality of the measurement was rated as *Good*, with minimal signal noise and reliable repeatability across replicates.

In contrast, the TiN sample (Figure S1b) exhibited a smaller average hydrodynamic diameter of 177.6 nm, with a higher PDI of 0.128, suggesting a broader size distribution. The main particle population had a peak at 120.9 nm, and the standard deviation was 43.72 nm. The result quality was also marked as *Good*, supporting the reliability of the data.

These findings indicate that while TiN particles are smaller on average, the hBN sample demonstrates superior uniformity in size distribution. Such differences may influence their respective performance in applications such as coating homogeneity or biological interactions, where particle size and uniformity play critical roles.

a)

			Size (d.n...	% Number:	St Dev (d.n...
Z-Average (d.nm):	230,3	Peak 1:	205,0	100,0	51,59
PdI:	0,012	Peak 2:	0,000	0,0	0,000
Intercept:	0,786	Peak 3:	0,000	0,0	0,000
Result quality	Good				

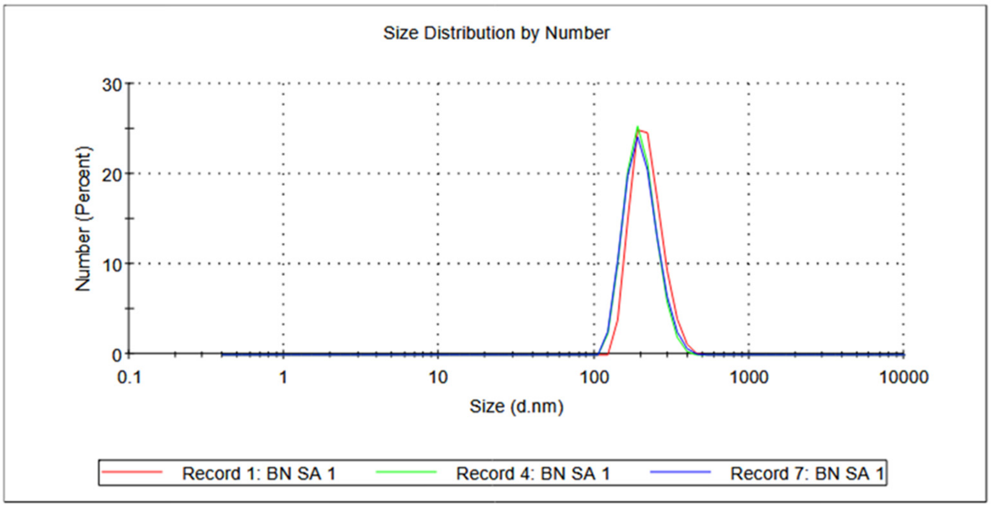

b)

			Size (d.n...	% Number:	St Dev (d.n...
Z-Average (d.nm):	177,6	Peak 1:	120,9	100,0	43,72
PdI:	0,128	Peak 2:	0,000	0,0	0,000
Intercept:	0,897	Peak 3:	0,000	0,0	0,000
Result quality	Good				

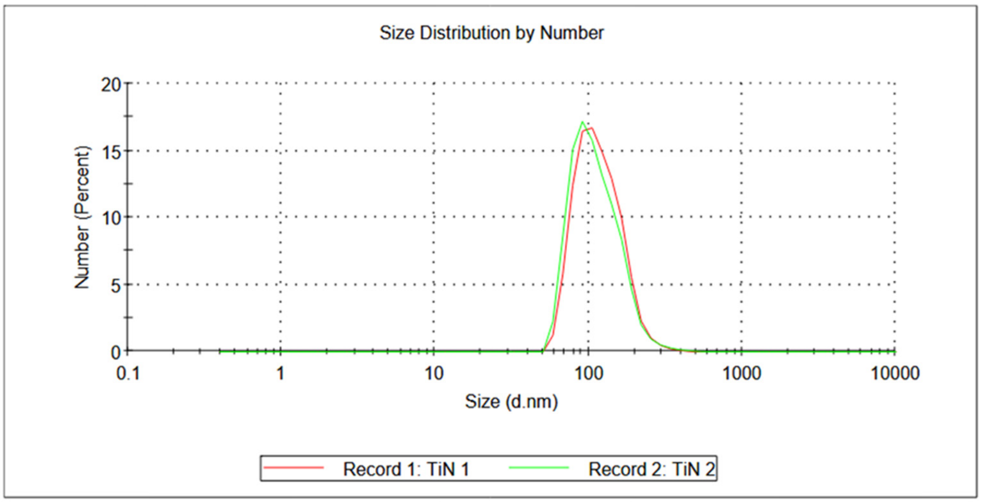

Figure S1. Dynamic Light Scattering (DLS) analysis showing particle size distribution by number for: (a) hBN and (b) TiN powder.
